# Tau seeding activity in various regions of down syndrome brain assessed by two novel assays

**DOI:** 10.1186/s40478-022-01436-2

**Published:** 2022-09-05

**Authors:** Nana Jin, Jianlan Gu, Ruozhen Wu, Dandan Chu, Yunn Chyn Tung, Jerzy Wegiel, Thomas Wisniewski, Cheng-Xin Gong, Khalid Iqbal, Fei Liu

**Affiliations:** 1 Department of Neurochemistry, Inge Grundke-Iqbal Research Floor, New York State Institute for Basic Research in Developmental Disabilities, Staten Island, NY 10314 USA; 2 Key Laboratory of Neuroregeneration of Jiangsu and Ministry of Education of China, Nantong University, Nantong, Jiangsu 226001 China; 3 Department of Developmental Neurobiology, New York State Institute for Basic Research in Developmental Disabilities, Staten Island, NY 10314 USA; 4 Center for Cognitive Neurology, Departments of Neurology, Pathology, and Psychiatry, NYU Grossman School of Medicine, New York, NY 10016 USA

**Keywords:** Down syndrome, Tau, Seeding activity, Phosphorylation

## Abstract

Propagation of tau pathology via the seeding of naive tau aggregation underlies the progression of Alzheimer’s disease (AD) and related tauopathies. Individuals with Down syndrome (DS) develop tau pathology at the fourth decade of life, but tau seeding activity in DS brain has not yet been determined. To measure tau seeding activity, we developed capture assay and seeded-tau aggregation assay with truncated tau_151-391_. By using brain extracts from AD and related tauopathies, we validated these two methods and found that the brain extracts from AD and related tauopathies, but not from controls and the diseases in which tau was not hyperphosphorylated, captured in vitro and seeded 3R-tau_151-391_ and 4R-tau_151-391_ to aggregate in cultured cells similarly. Captured tau_151-391_ levels were strongly correlated with the seeded-tau_151-391_ aggregation. Employing these two newly developed assays, we analyzed tau seeding activity in the temporal (TC), frontal (FC), and occipital cortex (OC); corpus callosum (CC); and cerebellar cortex (CBC) of DS and control brains. We found that the extracts of TC, FC, or OC, but not the CC or CBC of DS or the corresponding brain regions of control cases, captured tau_151-391_. Levels of the captured tau_151-391_ by brain extracts were positively correlated with their levels of phosphorylated tau. Extracts of cerebral cortex and CC, but not CBC of DS with a similar tau level, induced more tau_151-391_ aggregation than did the corresponding samples from the control cases. Thus, higher tau seeding activity associated with tau hyperphosphorylation was found in the TC, FC, and OC of DS compared with the corresponding control regions as well as with the CBC and CC of DS. Of note, these two assays are sensitive, specific, and repeatable at a low cost and provide a platform for measuring tau seeding activity and for drug screening that targets tau propagation.

**Supplementary Information:**

The online version contains supplementary material available at 10.1186/s40478-022-01436-2.

## Introduction

The accumulation and deposition of hyperphosphorylated tau aggregates in the brain is a hallmark of Alzheimer’s disease (AD) and neurodegenerative tauopathies, including corticobasal degeneration (CBD), progressive supranuclear palsy (PSP), and Pick’s disease (PiD). Tau pathology in AD brain is found sequentially in the trans-entorhinal–entorhinal areas, hippocampus, limbic areas, and finally in the associative and then primary neocortical areas, according to the Braak stages [4, 5]. It associates strongly with the progression of cognitive impairment [5], suggesting that regional propagation of tau pathology underlies the progression of AD. Recently, tau tracer retention measured by positron emission tomography (PET) showed similar stages [36, 51, 52].

The ability of misfolded tau seeds, that is, proteopathic tau, to recruit and template monomeric tau misfolding and propagate across brain regions has been widely studied and demonstrated in vitro and in vivo*. *In vitro, cytosolic and hyperphosphorylated tau isolated from AD brain (AD P-tau) sequesters normal tau to form filaments in a non-saturable manner [1, 2]. Pre-formed aggregates/filaments either generated in vitro or isolated from AD brain accelerate the aggregation of recombinant tau into paired helical filaments [16, 58]. In cultured cells, tau aggregates are internalized and induce aggregation of intracellular monomeric tau [17, 27, 38]. In tau transgenic mice, the inoculation of tau aggregates induces tau aggregation and the spread of tau pathology to distant brain regions [9, 10, 32, 34]. The seeding ability of tau from AD brains correlates positively with Braak stages and negatively with MMSE scores and precedes overt tau pathology [18]. In tau transgenic mice, tau seeds predict the spread of disease by appearing in brain regions prior to the appearance of any other pathological change [31]. Assessment of seeding activity of proteopathic tau in human samples may be relevant and may correlate with clinical data. In addition, tau seeding activity assays also provide a tool for drug screening that targets propagation of tau pathology.

Down syndrome (DS), caused by partial or complete trisomy of chromosome 21, is the most common chromosomal disorder and one of the leading causes of intellectual disability (ID). Today, as many as 6 million people worldwide are living with DS [54]. Individuals with DS develop AD pathology by the age of 40 years [62]. Imaging studies suggest a similar pattern of pathology between DS-AD and sporadic AD, but beginning at an earlier age in DS-AD. Tau burden assessed by PET in DS is similar to that in AD in binding pattern and progression [48]. Tau accumulation correlates with progressive neurodegeneration and cognitive decline, as do AD-specific hypometabolism and atrophy [48]. However, the seeding activity of proteopathic tau in the brain of individuals with DS has not been determined.

The seeding activity of proteopathic tau has been evaluated by seed amplification assay in vitro, cell-based assay, and in vivo seed amplification assay [40]. Tau in AD and most tauopathies is not mutated [22, 55]. Most tau seeding activity assays use tau with FTDP-17–associated mutations [40]. Here, by using truncated tau_151-391_, we report two new assays, an in vitro tau capture assay and a seeded-tau aggregation assay in cultured cells, for the assessment of tau seeding activity. We validated these two methods with brain extracts from AD, related tauopathies, and control cases and measured tau seeding activity in various regions of DS brain. The brain extracts from AD and related tauopathies captured tau in vitro and seeded-tau aggregation, but not from control brains or from the diseased brain tissues in which tau was not hyperphosphorylated. In DS brain, higher tau seeding activity in the temporal (TC), frontal (FC), and occipital cortex (OC) was found than in the corresponding regions of control brains. Extract of DS corpus callosum (CC) showed low tau seeding activity, but no detectable tau seeding activity, in DS cerebellar cortex (CBC). Tau seeding activity was positively correlated with the levels of hyperphosphorylated tau which displayed SDS- and β-mercaptoethanol-resistant high molecular weight (HMW) species. Of special note, these two methods can be performed using routine biochemical techniques. They can provide a platform for determining the role of post-translational modifications of tau in the captured tau and in the seeded-tau aggregates and for drug screening.

## Materials and methods

### Human brain tissue

Frozen tissue samples from the TC, FC, OC, CBC, and CC of DS and normal control brains (Table 1) were obtained from the Brain Bank for Developmental Disabilities and Aging of our institute. Diagnosis of DS trisomy 21 was extracted from subjects' medical records. Selection of control subjects was based on the diagnostic criteria developed at a consensus conference of the National Institute of Aging and the Reagan Institute. Cases with diagnosis of dementia, including AD and Parkinson disease were excluded. Cases with brain tumors, metastases, hemorrhages, multiple small infarcts, and brains with gross traumatic injury were also excluded. Exclusion of cases with Parkinson disease pathology was based on results of immunostaining for α-synuclein in Lewy bodies and neurites. Frozen autopsied tissues from FCs of five AD and five control brains (Table 2) were obtained from the Sun City Health Research Institute Brain Donation Program (Sun City, AZ, USA). The subjects were selected based on the criterion of diagnosis prior to death as being clinical AD. Control cases were cognitively normal, without any significant neuropathological findings that could contribute to cognitive symptoms. Frozen frontal cortical tissues from three CBD, three PiD, and two PSP brains (Table 3) were obtained from the Alzheimer’s Disease Research Center at New York University Grossman School of Medicine. The diagnosis of these cases was confirmed histopathologically by TW (a board-certified neuropathologist), using standard neuropathological criteria [3, 6, 30, 33, 39, 45, 57]. The use of autopsied frozen human brain tissue was in accordance with the National Institutes of Health guidelines and was exempted by the Institutional Review Board (IRB) of the New York State Institute for Basic Research in Developmental Disabilities because ‘‘the research does not involve intervention or interaction with the individuals’’ nor ‘‘is the information individually identifiable.’’ The brain tissue samples were stored at − 80 °C until used. Brain tissue was homogenized in cold buffer consisting of 20 mM Tris–HCl, pH 8.0, 0.32 M sucrose, 10 mM β-mercaptoethanol (β-ME), 5 mM MgSO_4_, 1 mM EDTA, 10 mM glycerophosphate, 1 mM Na_3_VO_4_, 50 mM NaF, 1 mM 4-(2-aminoethyl) benzenesulfonyl fluoride hydrochloride (AEBSF), and 10 μg/ml each of aprotinin, leupeptin, and pepstatin. After centrifugation of the homogenates at 10,000×*g* at 4°C for 10 min, the supernatants were used for analyses of seeding activity and phosphorylation of tau.

**Table 1:** Human brain tissues of Down syndrome and normal control used in this study

Group	Case #	Age (year)	Gender	PMI (h)	Braak stage^a^	FC	TC	OC	CC	CBC
DS	1342	61	M	3	VI	+		+	+	+
DS	1170	28	M	N/A	I		+			+
DS	375	43	F	N/A	V		+	+	+	
DS	1151	43	F	N/A	V	+	+	+	+	
DS	1335	47	F	N/A	VI	+		+	+	+
DS	1330	48	M	N/A	VI	+	+	+	+	
DS	1280	54	M	< 24	VI	+	+	+	+	+
DS	1238	55	M	6	VI	+	+	+	+	+
DS	1162	55	F	5	VI	+	+		+	+
DS	1308	57	F	< 24	VI	+	+	+	+	
DS	1139	58	F	5	VI	+	+	+	+	+
DS	1283	59	F	6	VI	+	+	+	+	+
DS	1322	59	M	< 24	VI	+	+	+	+	
DS	367	62	F	12	VI	+	+	+	+	+
DS	311	63	M	N/A	VI	+	+	+	+	
DS	709	63	M	NA	VI	+	+	+	+	+
DS	712	63	M	24	VI	+	+	+	+	+
DS	69	65	M	4.5	VI			+	+	+
DS	1153	65	M	N/A	VI	+		+	+	
DS	482	74	M	26	VI	+	+	+		+
Con	1169	32	M	14	N/A	+	+	+		
Con	247	31	M	3	N/A	+	+	+	+	+
Con	254	55	M	16.5	N/A	+	+	+	+	+
Con	256	59	M	6	N/A	+	+	+	+	+
Con	248	61	F	7	N/A	+	+	+	+	+
Con	255	67	F	4	N/A	+	+	+	+	+
Con	252	68	F	3	N/A	+	+		+	+
Con	241	68	F	2.5	N/A			+	+	
Con	596	71	M	7	N/A	+	+	+	+	+
Con	580	78	M	N/A	N/A	+			+	
Con	239	85	F	N/A	N/A	+	+	+	+	+
Con	244	86	M	1.5	N/A	+	+	+	+	+
Con	246	90	F	N/A	N/A	+	+	+	+	+

DS and control brain tissues were obtained from the Brain Bank for Developmental Disabilities and Aging of the New York State Institute for Basic Research in Developmental Disabilities

^a^Neurofibrillary pathology was staged according to Braak and Braak [5]. PMI, postmortem interval; FC, frontal cortex; TC, temporal cortex; OC, occipital cortex; CC, corpus callosum; CBC, cerebellar cortex

+ Tissues from various regions used in this study

**Table 2:** Human brain tissues of Alzheimer’s disease and control cases used in this study

Diagnosis	Case #	Age (year)	Gender	PMI (h)	Braak **s**tage^a^	Tangle score^b^	Disease duration (year)	Causes of death
AD	00–18	89	F	2.33	V	8.66	2	Stroke
AD	00–33	73	F	2	V	15	11	Dementia, Failure to thrive
AD	00–22	60	M	3.33	VI	15	8	Presenile dementia
AD	00–13	87	M	2.4	V	14.5	12	AD
AD	00–29	60	F	3.5	VI	15	9	Cardiac and/or respiratory
Con	00–34	85	M	3.16	II	4.25		Congestive heart failure
Con	03–28	80	M	2.16	I	1		Unknown
Con	03–50	91	M	3.33	III	3.5		Congestive heart failure
Con	03–63	83	F	3.25	II	0.75		Cerebrovascular accident
Con	00–49	86	F	2.5	III	5		Pulmonary fibrosis

Frontal cerebral cortices from AD and control cases were obtained from the Sun City Health Research Institute Brain Donation Program (Sun City, AZ, USA)

PMI, postmortem interval

^a^Neurofibrillary pathology was staged according to Braak and Braak [5]

^b^Tangle score was a density estimate and was designated none, sparse, moderate, or frequent (0, 1, 2 or 3 for statistics), as defined according to CERAD Alzheimer’s disease criteria [47]. Five areas (frontal, temporal, parietal, hippocampal, and entorhinal) were examined, and the scores were totaled for a maximum of 15

**Table 3:** Human brain tissues of corticobasal degeneration, Pick's disease, and progressive supranuclear palsy cases used in this study

Diagnosis	Case #	Age (Year)	Gender	PMI (h)	Pathology	Causes of death
CBD	TN10-34	81	F	3.5	CBD	Acute hypoxic respiratory failure secondary to pneumonia
CBD	TN09-27	84	M	12	CBD	Cardiac Arrest
CBD	TN15-09	95	M	11	CBD	Complications of inanition secondary to severe cognitive decline
PiD	TN15-83	83	F	12	FTLD-tau, A3, B3, C3, subacute infarctions	Complications of inanition secondary to severe cognitive decline
PiD	TN12-17	83	M	12	FTLD-tau, hippocampal sclerosis,A1, B1, C1	Complications of inanition secondary to severe cognitive decline
PiD	TN15-06	87	M	18	FTLD-tau, A0, B1, C0	Complications of inanition secondary to severe cognitive decline
PSP	TN15-85	95	M	15	PSP, A2, B2, C2	Asphyxia secondary to aspiration episode
PSP	TW19-31	72	M	16	PSP, A3, B3, C3, right frontal lobe remote infarction	Complications of inanition secondary to severe cognitive decline

Frozen brain tissues were obtained from the New York University Alzheimer’s Disease Research Center

PMI, postmortem interval; CBD, corticobasal degeneration; PiD, Pick's disease; FTLD-tau, frontotemporal lobar degeneration with tau pathology; PSP, progressive supranuclear palsy

### Plasmids, antibodies, and other reagents

pCI/HA-tau_1-441_, pCI/HA-tau_151-391_, pCI/HA-Trans-active response DNA-binding protein-of 43 (TDP-43)_1–414_, and pCI/HA-TDP-43_100–414_ were constructed as described previously [25, 26]. The primary antibodies used in the present study are listed in Table 4. Horseradish peroxidase (HRP)-conjugated anti-mouse and anti-rabbit IgGs were obtained from Jackson ImmunoResearch Laboratories (West Grove, PA, USA). Alexa 555- and Alexa 488-conjugated-secondary antibodies were from Thermo Fisher Scientific corporation (Waltham, MA, USA).

**Table 4:** Primary antibodies used in the present study

Antibody	Type	Species	Specificity	Source/reference (catalog)
43D	Mono-	M	Tau (8–16)	In-house/BioLegend (816,601)[42]
77G7	Mono-	M	Tau (316–355)	In-house/BioLegend (816,701)[42]
R134d	Poly-	R	Total tau	In-house[42]
AT8	Mono-	M	pSer202/Thr205-tau	Thermo Scientific (MN1020)
Anti-pS199	Poly-	R	pSer199-tau	Invitrogen (44-734G)
Anti-pT205	Poly-	R	pThr205-tau	Invitrogen (44-738G)
Anti-pT212	Poly-	R	pThr212-tau	Invitrogen (44-740G)
Anti-pS214	Poly-	R	pSer214-tau	Invitrogen (44-742G)
Anti-pT217	Poly-	R	pSer217-tau	Invitrogen (44–744)
AT180	Mono-	M	pThr231-tau	Invitrogen (MN1040)
12E8	Mono-	M	pSer262/Ser356-tau	Dr. D. Schenk
PHF-1	Mono-	M	pSer396/Ser404-tau	Dr. Peter Davies [23]
R145d	Poly-	R	pSer422-tau	In-house [42]
Anti-HA	Mono-	M	HA	Sigma (H9658)
RD3	Mono-	M	3R-tau	Millipore (05–803)
Anti-GAPDH	Poly-	R	GAPDH	Sigma (G9545)

*Mono* monoclonal, *Poly* polyclonal, *p* phosphorylated, *M* mouse, *R* rabbit

### Cell culture and transfection

Human embryonic kidney cell line (HEK-293FT) and human cervix epithelia cell line (HeLa) were cultured in Dulbecco’s modified Eagle’s medium (DMEM) (Thermo Fisher Scientific) supplemented with 10% fetal bovine serum (FBS) (Invitrogen), 100 U/ml penicillin, and 100 μg/ml streptomycin (Thermo Fisher Scientific) and incubated in a humidified atmosphere containing 5% CO_2_ at 37 °C. Cells were seeded to culture plates, and all transfections were performed with FuGENE HD (Promega, Madison, WI, USA) or Lipofectamine™ 2000 (Thermo Fisher Scientific) according to the manufacturer’s instructions. Empty vectors were used as controls for the corresponding transfection.

### Preparation of oligomeric tau from AD brain

Oligomeric tau derived from AD brain (AD O-Tau) was isolated from the cerebral cortex of frozen autopsied AD brains, as we described previously [32, 43]. Briefly, 10% brain homogenate prepared in the buffer (20 mM Tris–HCl, pH 8.0, 0.32 M sucrose, 10 mM β-ME, 5 mM MgSO_4_, 1 mM EDTA, 10 mM glycerophosphate, 1 mM Na_3_VO_4_, 50 mM NaF, 1 mM AEBSF, and 10 μg/ml each of aprotinin, leupeptin, and pepstatin) was centrifuged at 27,000×*g* for 30 min. The supernatant was further centrifuged at 235,000×*g* for 45 min, and the resulting pellet, i.e., AD O-Tau–enriched fractions, was collected, washed three times, and then resuspended in normal saline. The AD O-Tau was probe-sonicated for 10 min at 20% power and stored at − 80 °C till used.

### Western Blot and immuno-dot blot

Western blot: Brain extracts were adjusted to 1 × Laemmli sample buffer, followed by heating in a boiling-water bath for 5 min. Cultured cells were lysed directly in the Laemmli sample buffer containing 1 mM AEBSF and 10 μg/ml each of aprotinin, leupeptin, and pepstatin and then heated as above. Protein concentration was determined using the Pierce™ 660 nm Protein Assay kit (Thermo Fisher Scientific, Waltham, MA, USA). Samples were subjected to SDS-PAGE and transferred onto a polyvinylidene fluoride membrane (MilliporeSigma, Burlington, MA, USA). The membrane was subsequently blocked with 5% fat-free milk in Tris-buffered saline (TBS) for 30 min, incubated with primary antibody (Table 4) diluted in 5% fat-free milk in TBS containing 0.1% NaN_3_ overnight at room temperature (RT), washed with TBST (TBS with 0.05% Tween 20) three times, and incubated with HRP–conjugated secondary antibody for 2 h at RT, washed with TBST, incubated with the enhanced chemiluminescence (ECL) substrate (Thermo Fisher Scientific), and exposed to HyBlot CL® autoradiography film (Denville Scientific Inc., Holliston, MA, USA) or detected by iBright Imager (Thermo Fisher Scientific). Alternatively, the membrane incubated with Alexa 488- or Alexa 555- conjugated secondary antibody for 2 h at RT, washed with TBST and detected by iBright Imager (Thermo Fisher Scientific). Specific immunosignal was quantified by using the Multi Gauge software V3.0 from Fuji Film (Minato, Tokyo, Japan).

Immuno-dot blot: Various amounts of samples were applied onto a nitrocellulose (NC) membrane (Schleicher and Schuell, Keene, NH, USA) at 5 μl per grid of 7 × 7 mm size in duplicates or triplicates. The membrane was placed in a 37 °C oven for 1 h to allow the protein to bind to the membrane. The membrane was subsequently blocked and incubated with primary antibody and then with secondary antibody as described above for Western blots.

### Tau capture assay for measuring tau seeding activity in vitro

Cell lysate containing hemagglutinin-tau (HA-tau): HEK-293FT cells were transfected with pCI/HA-tau_151-391_ (in the numbering of the 441–amino acid isoform of human tau) or pCI/HA-tau_1-441_ for 48 h. To obtain cell lysate containing TDP-43, HEK-293FT cells were transfected with pCI/HA-TDP-43_1–414_ similarly. The cells were washed with cold-phosphate buffered saline (PBS) and probe-sonicated in cold lysate buffer (50 mM Tris–HCl, pH7.4, 0.15 M NaCl, 1 mM EDTA, 1 mM Na_3_VO_4_, 50 mM NaF, 1 mM AEBSF and 10 μg/ml each of aprotinin, leupeptin, and pepstatin) for 2 min with 20% amplitude at 4 °C 48 h after transfection. Cell lysate was centrifuged for 5 min at 10,000×*g*. The level of tau in the cell lysates was analyzed by immuno-dot blots, and lysates were stored at −80 °C until used.

Capture of tau from the cell lysate: Various amounts of AD O-tau or brain extracts were applied onto a NC membrane as described in immuno-dot blot. The membrane was blocked with 5% fat-free milk in TBS for 30 min and incubated with cell lysates containing HA-tau or HA-TDP-43 overnight at RT. After washing, the membrane was developed with anti-HA followed by incubation with HRP-conjugated secondary antibody and ECL as described above for Western blots to detect captured tau.

### Seeded-tau aggregation assay for assessing tau seeding activity in cultured cells

HEK-293FT cells cultured in 24-well plate were transfected with pCI/HA-tau_151–391_, pCI/HA-tau_1-441_ or pCI/HA-TDP-43_100–414_ with FuGENE HD. Six hr after transfection, the cells were treated with AD O-tau or brain extracts in 25 μl of Opti-MEM containing 3% Lipofectamine 2000 for 42 h and then lysed in RIPA buffer (50 mM Tris–HCl, pH7.4, 150 mM NaCl, 1% Nonidet P-40, 0.5% sodium deoxycholate, and 0.1% SDS) containing 50 mM NaF, 1 mM Na_3_VO_4_, 1 mM AEBSF, 5 mM benzamidine, and 10 μg/ml each of aprotinin, leupeptin, and pepstatin for 20 min on ice. The cell lysates were centrifuged at 75,000×*g* for 30 min. The supernatant was saved as RIPA buffer–soluble fraction, and the pellet, RIPA buffer–insoluble fraction, was washed with PBS. Levels of RIPA buffer–insoluble and -soluble Tau or TDP-43 were analyzed by Western blots developed with anti-HA.

To visualize tau aggregates in cells, HeLa cells were transfected to express HA-tau_1–441_ or HA-tau_151–391_ and treated with AD O-Tau for 42 h as described above. The cells were then fixed for 15 min with 4% paraformaldehyde in phosphate buffer, washed with PBS, and treated with 0.3% Triton X-100 in PBS for 15 min at RT. After blocking with 5% newborn goat serum, 0.1% Triton X-100, and 0.05% Tween 20 in PBS for 30 min, the cells were incubated with anti-HA in blocking solution overnight at 4 °C, washed with PBS, and incubated with Alexa 488–conjugated secondary antibody for 2 h at RT. After washing with PBS, the cells were mounted with ProLong^TM^ Gold antifade reagent (Thermo Fisher Scientific) and observed with a Nikon confocal microscope.

### Depletion of tau from AD brain extract

Tau antibodies 77G7 and a mixture of 43D and HT7, and as a control, mouse IgG (mIgG) were incubated with protein G-agarose for 6 h at RT. After washing with TBS, the antibodies-coupled beads were incubated with the same volume of AD brain extract overnight at 4 °C. The supernatant was saved as tau-depleted AD extract for further analysis.

### Guanidine hydrochloride and urea treatment

AD O-tau was dotted on a NC membrane. The membrane was treated with 6 N guanidine hydrochloride (GuHCl), 8 M urea, or TBS as control for 2 h, followed by tau capture assay, described above.

### Statistical analysis

GraphPad Prism 6 (GraphPad Software Inc., San Diego, CA, United States) was used for statistical analysis. Comparison between two groups was analyzed by unpaired two-tailed Student’s *t* test (for data with normal distribution) or Mann–Whitney test (for data with non-normal distribution). One-way or two-way analysis of variance (ANOVA) followed by Tukey’s or by Sidak’s multiple comparisons was used in this study. Data was presented as mean ± standard deviation (SD). For correlation analysis, linear or non-linear regression correlation coefficient was calculated. For linear regression, Pearson (for data with normal distribution) or Spearman (for data with non-normal distribution) correlation was performed. *p* < 0.05 was considered statistically significant.

## Results

### Tau capture assay assesses the seeding activity of AD O-tau in vitro

**Fig. 1:**
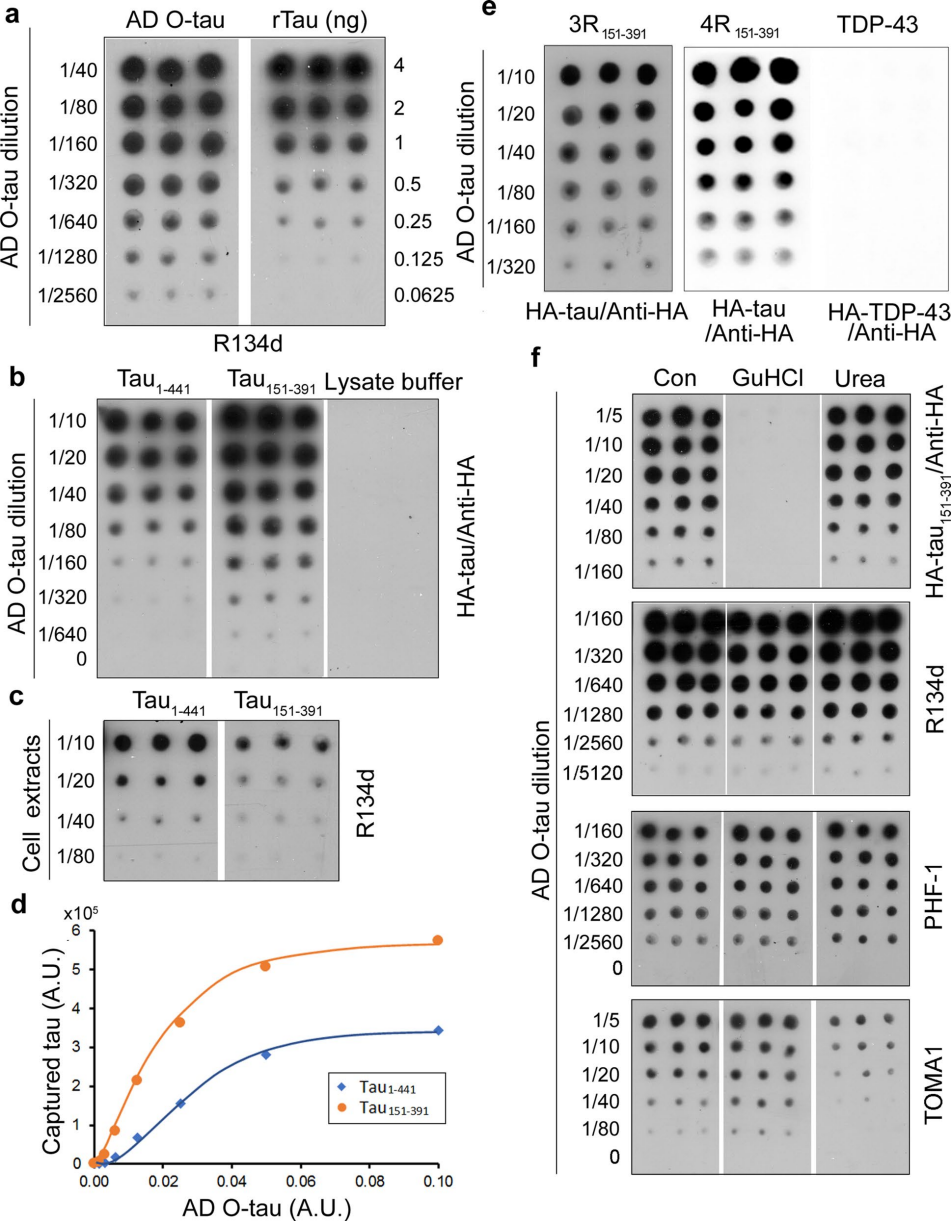
Tau capture assay assessed the seeding activity of AD O-tau. **a** Tau level in AD O-tau was measured by immuno-dot blot. Various amounts of AD O-tau and tau39 (2N3R tau) were applied onto nitrocellulose (NC) membrane and developed with pan-tau antibody R134d. **b**–**d**. Tau_151-391_ was effectively captured by AD O-tau. Various amounts of AD O-tau were applied onto NC membrane and incubated with HEK-293FT cell extracts containing full-length (tau_1-441_) or truncated (tau_151-391_) tau40 (2N4R tau) tagged with HA. Captured HA-tau was developed with anti-HA followed with HRP-anti-mouse IgG (**b**). The level of tau in the cell lysates was analyzed by immuno-dot blots developed with anti-HA (**c**). The mean levels of captured tau_1-441_ or tau_151-391_ were plotted against the relative levels of AD O-tau (**d**). A.U., arbitrary unit. **e** AD O-tau could not recruit TDP-43 from cell lysate. NC membranes pre-applied with AD O-tau were incubated with 3R-tau_151-391_ or 4R-tau_151-391_ and TDP-43_1–414_, followed with anti-HA and HRP-anti-mouse IgG. **f** Guanidine hydrochloride (GuHCl) killed the seeding activity of AD O-tau. NC membranes pre-applied with AD O-tau were treated with TBS, 6 N GuHCl, or 8 M urea for 2 h at RT. After washing with TBS, the membranes were subjected to tau capture assay or immuno-dot blots developed with R134d (pan-tau), PHF-1 (phospho-tau at Ser396/404), and TOMA1 (oligomeric tau)

Proteopathic tau seeds recruit and convert naïve tau to disease-associated, higher-order structures in a prion-like fashion. Based on the prion-like property, we developed a tau capture assay to assess tau seeding activity by using AD O-tau as proteopathic tau seeds. Previous studies showed that the AD O-tau fraction effectively templated tau aggregation in vitro and in vivo [32, 43]. Tau in the AD O-tau fraction was quantified by immuno-dot blots developed with pan-tau antibody R134d, in which recombinant human tau (rTau) was used as a standard (Fig. 1a). Tau in AD and most other tauopathies is not mutated. We previously found that deletion of N-terminal 150 a.a. and C-terminal 50 a.a. showed the strongest effect on enhancing tau binding to AD O-tau and aggregation seeded by AD O-tau [26]. To determine the seeding activity of AD O-tau, we applied various amounts of AD O-tau on a NC membrane. The membrane was incubated overnight at RT with cell lysate containing HA tagged as either full-length tau (HA-tau_1-441_) or truncated tau (HA-tau_151-391_). Captured tau was analyzed by developing the membrane with anti-HA (Fig. 1b). We found that both HA-tau_1-441_ and HA-tau_151-391_ were recruited from the cell lysates by AD O-tau dose-dependently, but much more tau_151-391_ than tau_1-441_ was captured (Fig. 1b,d), even when the expression level of tau_151-391_ in the cell lysate was less than tau_1-441_ (Fig. 1c). The sensitivity of the capture assay by using HA-tau_151-391_ was approximately ~ 1 ng tau in AD O-tau fraction (Fig. 1c), as estimated by using the rTau standard (Fig. 1a). Thus, we employed HA-tau_151-391_ to perform the capture assay.

Neurofibrillary tangles (NFTs) in AD brain contain tau isoforms with three or four microtubule-binding repeats, 3R-tau and 4R-tau, respectively, resulting from alternative splicing of tau exon 10 [8, 19]. We found that AD O-tau also effectively recruited 3R-tau_151-391_ and 4R-tau_151-391_ in a dose-dependent manner (Fig. 1e), but not TDP-43 (Fig. 1e), indicating that TDP-43 cannot be cross-captured by tau seeds and that this tau capture assay is specific for proteopathic tau seeds.

Proteopathic tau seeds multiply through a process of self-propagation, where the β-sheet acts as a template for the formation of nascent aggregates [63]. We previously showed that boiling did not affect AD O-tau to recruit tau from cell lysate [43]. Urea and GuHCl are widely used to denature the protein that inactivates prions [7, 49]. We applied various amounts of AD O-tau on NC membranes. The membranes were treated with 6 M GuHCl, 8 M urea in TBS, or TBS as a control, for 2 h and were then subjected to the capture assay or dot-blots developed with R134d (pan-tau), PHF-1 (phospho-tau at Ser396/404), or TOMA1 (oligomeric tau). We found that neither GuHCl nor urea affected or only slightly affected the immunoreactivity of AD O-tau toward R134d, PHF-1 or TOMA1. However, GuHCl treatment almost completely abolished the ability to capture tau, but urea did not show any obvious effects (Fig. 1d). Thus, tau seeding activity can be killed by denaturants selectively.

### Seeded-tau aggregation assay in cultured cells evaluates the seeding activity of AD O-tau

**Fig. 2:**
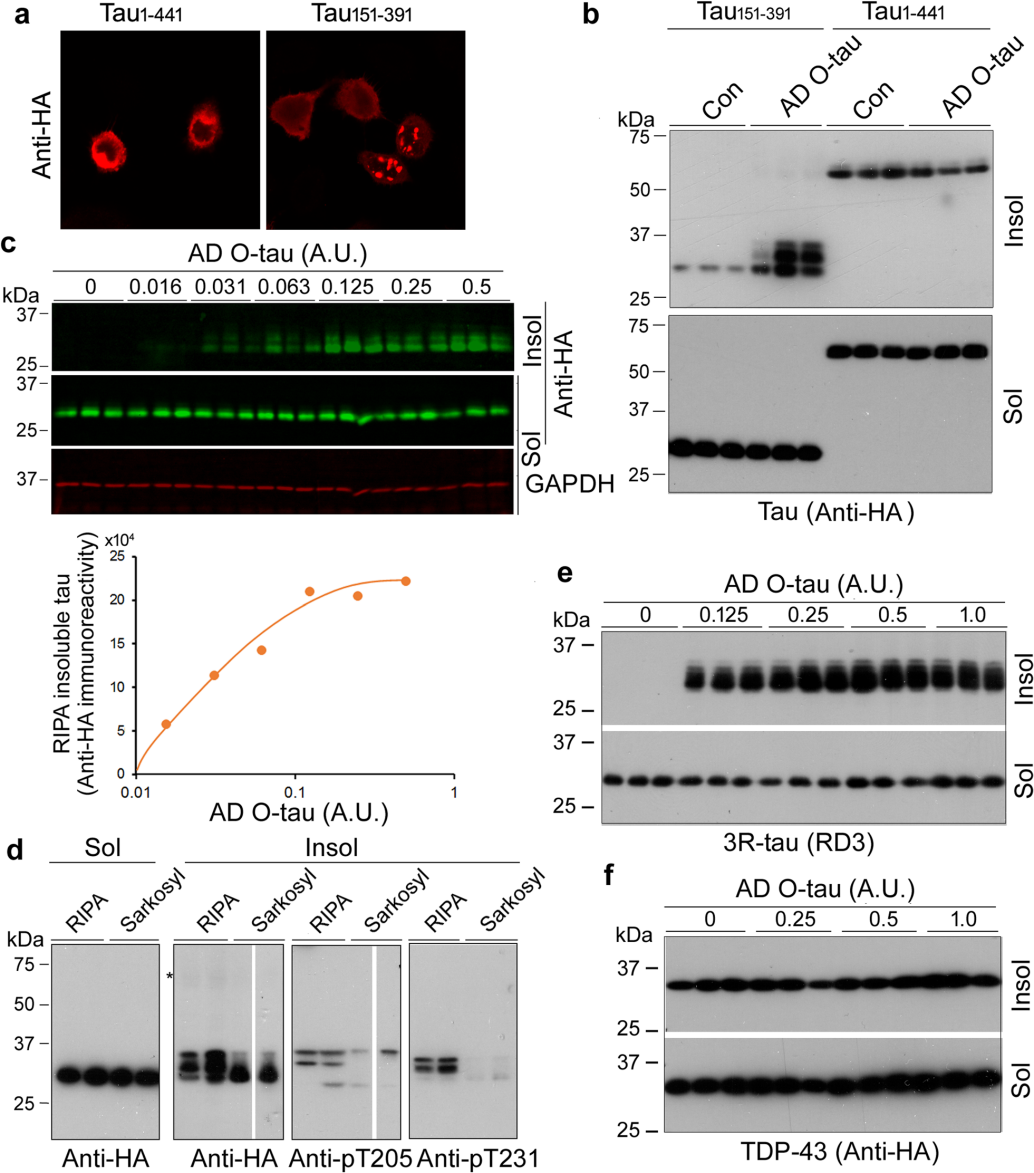
Seeded-tau aggregation assay in cultured cells to evaluate the seeding activity of AD O-tau. **a** AD O-tau seeded tau_151-391_ aggregation in HeLa cells. HeLa cells were transfected with pCI/HA-tau_1-441_ or pCI/HA-tau_151-391_, treated with AD O-tau for 42 h after 6 h tau transfection, and immunostained with anti-HA. **b** AD O-tau seeded tau_151-391_ aggregation in HEK-293FT cells. HEK-293FT cells were transfected with pCI/HA-tau_1-441_ or pCI/HA-tau_151-391_ and treated with AD O-tau, as described in panel A. The cells were lysed with RIPA buffer. RIPA-insoluble and -soluble fractions yielded by ultracentrifugation were analyzed with Western blots developed with anti-HA. **c** AD O-tau induced tau_151-391_ aggregation dose-dependently in cultured cells. HEK-293FT cells overexpressing HA-tau_151-391_ were treated with various amounts of AD O-tau for 42 h. The RIPA-soluble and -insoluble tau were analyzed by Western blots. The levels of RIPA-insoluble tau were plotted against various amounts of AD O-tau. **d** Phosphorylation of RIPA-insoluble tau was different from that of sarkosyl-insoluble tau. HEK-293FT cells overexpressing HA-tau_151-391_ were treated with AD O-tau. The cells were lysed with RIPA-buffer or 1% Sarkosyl buffer. The insoluble and soluble tau were analyzed by Western blots developed with anti-HA or with anti-phospho-tau (pT205- or pT231-tau). **e**, **f** AD O-tau seeded 3R-tau_151-391_, but not TDP-43, aggregation. HEK-293FT cells expressing HA-3R-tau_151-391_ or HA-TDP-43_100–414_ were treated with various amounts of AD O-tau for 42 h. RIPA-insoluble and -soluble 3R-tau_151-391_ (**e**) or TDP-43_100–414_ (**f**) were analyzed by Western blots developed with anti-3R-tau (RD3) or anti-HA

We previously reported that tau_151-391_ can form aggregates most effectively when seeded by AD O-tau [26]. To confirm tau_151-391_ aggregation seeded by AD O-tau, we overexpressed tau_1-441_ and tau_151-391_ in HeLa or HEK-293FT cells and treated them with AD O-tau for 42 h after 6 h cell transfection. Then, the HeLa cells were immunostained with anti-HA. We found tau-aggregated puncta in HeLa/tau_151-391_ cells treated with AD O-tau (Fig. 2a). In a previous study we showed that AD O-tau seeded tau_151-391_ aggregates are thioflavin T-positive [26]. Amyloid-type protein aggregates are detergent-insoluble. For biochemical analysis of aggregated tau, we lysed the HEK-293FT cells with RIPA buffer. RIPA-insoluble and -soluble tau were analyzed by Western blots developed with anti-HA. We found RIPA-insoluble tau in HEK-293FT/tau_151-391_ cells treated with AD O-tau (Fig. 2b), indicating that AD O-tau effectively seeded tau_151-391_ aggregation.

To learn whether seeded tau_151-391_ aggregation assay can be used to measure seeding activity, we treated HEK-293FT/tau_151-391_ cells with various amounts of AD O-tau for 42 h and analyzed RIPA-insoluble and -soluble taus by Western blots. We found an increase of RIPA-insoluble tau that was dose-dependent (Fig. 2c), suggesting that seeding activity of AD O-tau can be evaluated by the seeded-tau aggregation assay in cultured cells.

Sarkosyl solution was widely used to separate aggregated tau from brain tissues in previous studies. We lysed the AD O-tau–treated HEK-293FT/tau_151-391_ cells with RIPA buffer and 1% sarkosyl in buffer and analyzed the soluble and insoluble tau. We found similar levels and patterns of soluble tau in both treatments (Fig. 2d), but higher levels of phosphorylated tau in the RIPA-insoluble fractions than the sarkosyl-insoluble fractions (Fig. 2d). These results suggest that RIPA-buffer–insoluble tau may present aggregated tau better because aggregated tau in AD is hyperphosphorylated [35].

AD O-tau contains both 3R-tau and 4R-tau. We determined that AD O-tau seeded 3R-tau aggregation in HEK-293FT/3R-tau_151-391_ cells. We found that AD O-tau templated 3R-tau aggregation dose-dependently (Fig. 2e). However, no increased RIPA-insoluble TDP-43 was observed (Fig. 2f), further confirming that TDP-43 cannot be cross-seeded by tau seeds and that the seeded-tau aggregation assay is specific for tau seeds.

### The brain extracts of AD and related tauopathies captures and seeds 3R- and 4R-tau_151-391_ aggregation similarly

**Fig. 3:**
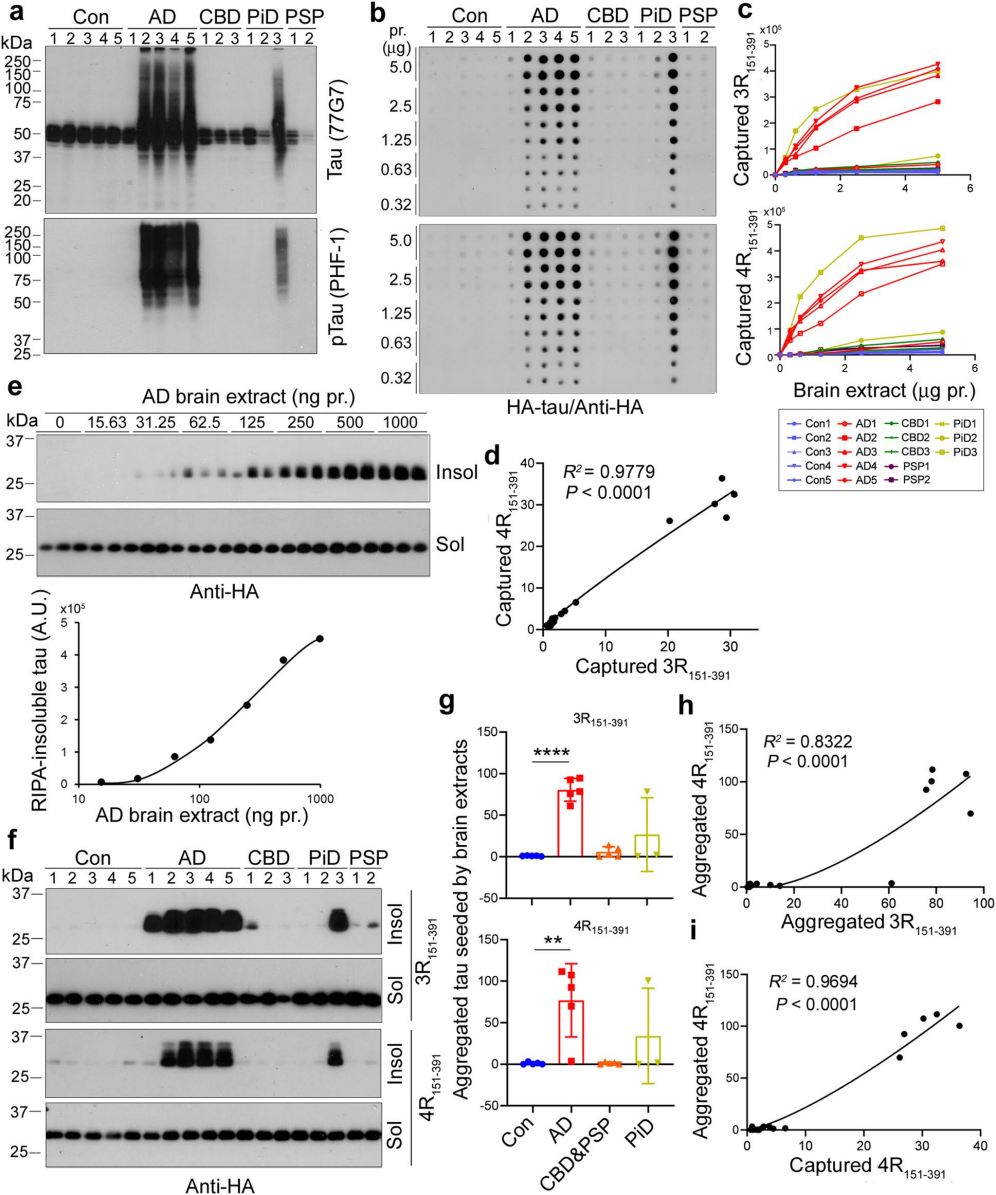
Tau seeding activity in the brains of AD and related tauopathies determined by capture and seeded-tau aggregation assays. **a** Tau is hyperphosphorylated in the brains of AD and related tauopathies. Tau and phospho-tau in the brain extracts of frontal cortices from five AD, five control, three CBD, three PiD, and two PSP cases were analyzed by Western blots developed with 77G7 (pan-tau) and PHF-1 (pS396/404-tau). **b**–**d** 3R-tau and 4R-tau were captured by brain extracts from AD and related tauopathies in which tau was hyperphosphorylated. Various amounts of brain extracts were applied onto a NC membrane. The membrane was incubated with cell extracts containing HA-3R-tau_151-391_ or HA-4R-tau_151-391_ for capture assay (**b**). The levels of captured tau were plotted against protein amounts in the brain extracts dotted on the membrane (**c**). Levels of captured 4R-tau_151-391_ were plotted against levels of captured 3R-tau_151-391_. The correlation was analyzed by non-linear regression (**d**). **e** AD brain extract seeded tau_151-391_ aggregation dose-dependently. HEK-293FT cells expressing HA-3R-tau_151-391_ were treated with various amounts of AD brain extracts for 42 h. RIPA-insoluble and -soluble taus were analyzed by Western blots. The levels of RIPA-insoluble tau (HA immunoreactivity) were plotted against protein amounts of AD brain extracts. **f**–**h** Aggregation of 3R-tau and 4R-tau were seeded similarly by the brain extracts of AD and other tauopathies in which tau was hyperphosphorylated. HEK-293FT cells expressing HA-3R-tau_151-391_ or HA-4R-tau_151-391_ were treated with brain extracts from five AD, five control, three CBD, three PiD, and two PSP cases for 42 h. RIPA-insoluble and -soluble tau were analyzed by Western blots (**f**). The levels of RIPA-insoluble tau are presented as mean $\pm$ SD, with each dot representing an individual brain sample (**g**). **P < 0.05; ****P < 0.0001. Correlation of RIPA-insoluble 3R-tau_151-391_ and 4R-tau_151-391_ seeded by the brain extracts was analyzed by non-linear regression (**h**). **i** Tau seeding activity determined by capture assay was consistent with that determined by seeded-tau aggregation assay. Correlation of RIPA-insoluble 4R-tau_151-391_ seeded and 4R-tau_151-391_ captured by the brain extracts was analyzed by non-linear regression. Although all tauopathies are characterized by tau pathology of hyperphosphorylated and seeding competent tau, we did not detect it in the tissue pieces used from several cases, suggesting that the pathology is not evenly distributed throughout the frontal cortex in tauopathies

To determine whether these two assays can evaluate tau seeding activity in AD and related tauopathies’ brain, various amounts of brain extracts of frontal cortices from five AD, five control, three CBD, three PiD, and two PSP cases were obtained by 10,000 xg for 10 min, which contained seeding-competent tau [56, 64]. We first analyzed the levels of tau and phosphorylated tau in these tissue samples by Western blots developed with antibodies 77G7 (pan-tau) and PHF-1 (phospho-tau at Ser396/404) (Fig. 3a) and found hyperphosphorylation of tau at Ser396/404, which also displayed SDS- and β-ME resistant HMW-species, the common features of AD and related tauopathies, in AD2-5 and PiD3, but not in Con1-5, AD1, CBD1-3, PiD1,2, and PSP1,2 brains (Fig. 3a), suggesting that the pathology in these cases is not evenly distributed throughout the frontal cortex in tauopathies.

To measure tau seeding activity in these brain tissues and to determine whether brain extracts of AD and related tauopathies can recruit both 3R-tau and 4R-tau, we performed the tau capture assay. We applied various amounts of the brain extracts onto NC membranes and incubated the membranes with the HEK-293FT cell lysates containing HA-3R-tau_151-391_ or HA-4R-tau_151-391_. Captured tau was developed with anti-HA. We found that both 3R- and 4R-tau_151-391_ were captured from the corresponding cell lysates dose-dependently by brain extracts of AD and PiD3, but not from those tissue samples that did not contain hyperphosphorylated and HMW-tau species, including control and one AD and seven tauopathy cases (Fig. 3a–c). We found that 0.32 μg protein of AD brain extract, which contained ~ 4 ng tau, was effective to recruit tau_151-391_ (Fig. 3b, c). Depletion of tau by 43D & HT7 significantly reduced the ability of AD brain extract to capture tau (Additional file 1: Fig. S1a, b). In an independent experiment, we found that tau_151-391_ was captured similarly by the AD brain extracts, but not by brain extracts from controls (Additional file 1: Fig. S2a). Thus, the capture assay is specific, sensitive, and repeatable for measuring tau seeding activity.

Tau pathology in AD contains both 3R-tau and 4R-tau, but only 4R-tau is found in CBD and PSP, and mainly 3R-tau is found in PiD [21]. To compare the capture ability towards 3R-tau and 4R-tau, a linear regression was carried out, showing a positive correlation between levels of captured 3R-tau and 4R-tau by the brain extracts (Fig. 3d). Surprisingly, we observed that the slope was ~ 1, indicating that AD and other related tauopathies’ brain extracts recruit 3R-tau and 4R-tau equally.

To assess the seeding activity of the brain extracts by seeded-tau aggregation assay in cultured cells, we treated HEK-293FT/HA-3R-tau_151-391_ with various amounts of AD brain (case 2) extract for 42 h and analyzed RIPA-insoluble and -soluble tau with Western blots. We found similar RIPA-soluble tau in the cells treated with various amounts of AD brain extract, but increased RIPA-insoluble tau that was induced by AD brain extract in a dose-dependent manner. The sensitivity of the assay was ~ 31.25 ng protein of AD brain extract (Fig. 3e). Depletion of tau with 43D&HT7 significantly reduced tau_151-391_ aggregation seeded by AD brain extract in cultured cells (Additional file 1: Fig. S1c,d).

To detect tau seeding activity in AD and related tauopathies’ brain extracts above by seeded-tau aggregation assay, we treated HEK-293FT/HA-3R-tau_151-391_ or HA-4R-tau_151-391_ cells with these brain extracts for 42 h and analyzed RIPA-insoluble and -soluble tau (Fig. 3f). We found both insoluble 3R- and 4R-tau_151-391_ in the cells treated with AD2-3 and PiD3 brain extracts, but not in the cells treated with brain extracts from cases that contained no detectable hyperphosphorylated and HMW-tau species (Fig. 3f,g). In an independent experiment, similar 4R-tau_151-391_ aggregation was seeded by AD and control brain extracts, indicating high repeatability of the assay (Additional file 1: Fig. S2b). Furthermore, seeded-3R-tau aggregates were positively correlated to seeded-4R-tau aggregates (Fig. 3h), confirming that proteopathic tau in AD and related tauopathies’ extracts induced both 3R-tau and 4R-tau aggregation similarly.

To learn the relationship between tau seeding activity detected by the tau capture assay and by the seeded-tau aggregation assay, we plotted tau levels captured against aggregated tau levels seeded by the brain extracts above and analyzed by non-linear regression. We found that the levels of captured tau strongly correlated with the levels of seeded tau aggregates, and the *R*^2^ was 0.9694 (Fig. 3i). These results suggest that both the tau capture assay and the seeded-tau aggregation assay are sensitive, specific, and repeatable. Both assays are consistent in measuring tau seeding activity, but the seeded-tau aggregation assay is 10 times more sensitive than the tau capture assay.

### Tau seeding activity in various brain regions of individuals with DS assessed by the tau capture assay

**Fig. 4:**
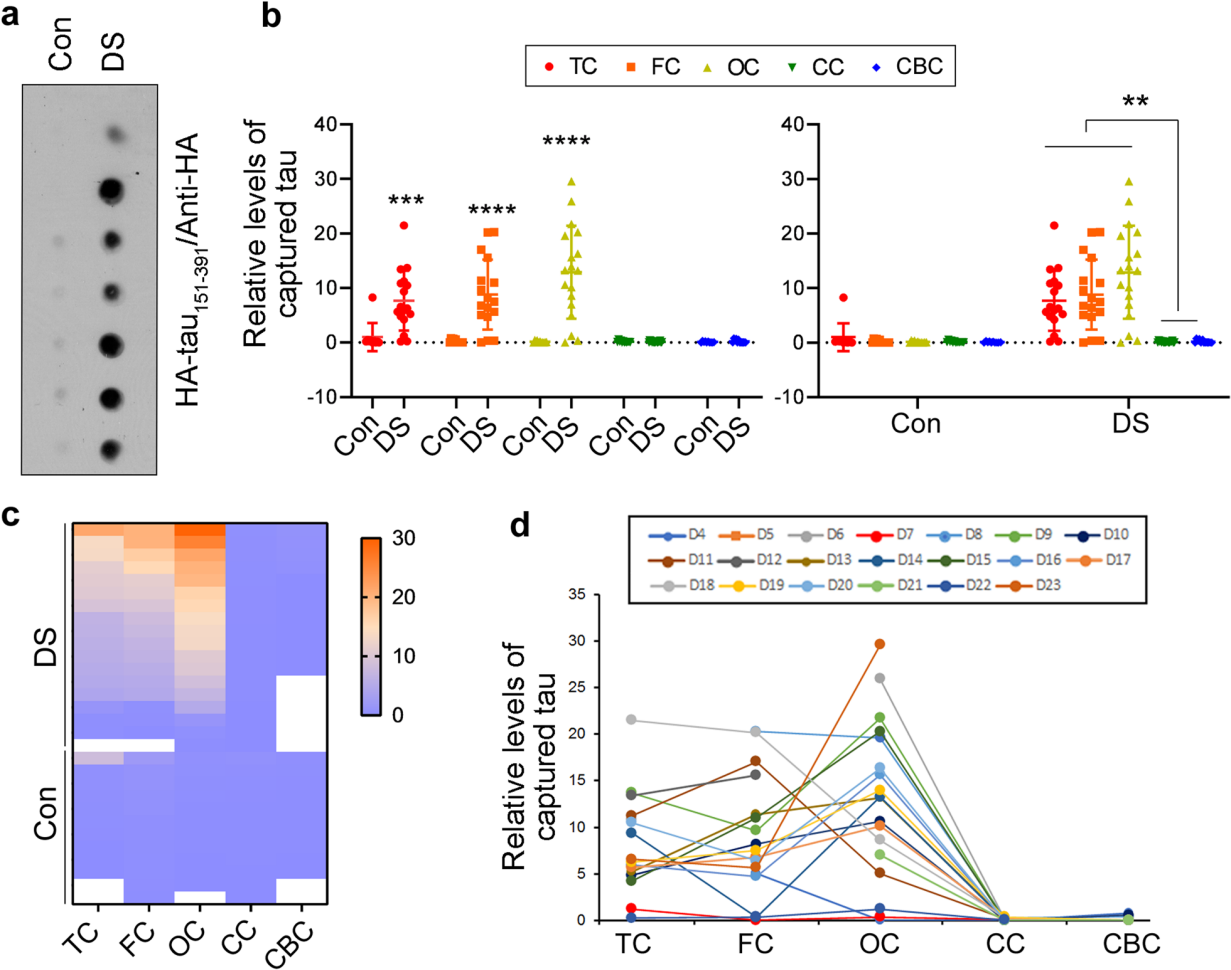
Seeding activity of proteopathic tau in DS brain extracts found by tau capture assay. **a**. Representative image of tau capture assay. Brain extracts of temporal cortex (TC), frontal cortex (FC), occipital cortex (OC), corpus callosum (CC), and cerebellar cortex (CBC) from DS and control cases were applied onto a NC membrane in the same amount of protein and subjected to tau capture assay by incubating the membrane with HEK-293FT/HA-tau_151-391_ cell lysate. The captured tau was developed with anti-HA followed by HRP-2^nd^-Ab and ECL. **b**, **c**. The levels of captured tau are presented as a scatter plots with mean ± SD (**b**) and heatmap (**c**). **P < 0.01; ***P < 0.001; ****P < 0.0001. **d**. Seeding activity crossing various brain regions assessed by tau capture assay in DS cases

Individuals with DS develop Alzheimer-type tau pathology in the 4th decade of life [62]. AD brain displays region-specific tau seeding activity [37]. However, the regional tau seeding activity of DS brain is not known. We determined tau seeding activity in various regions of DS brain by tau capture assays. We applied the same amount of protein of extracts from various regions of DS brain onto a NC membrane and carried out the tau capture assay (Fig. 4a). We found that the TC, FC, and OC, but not the CC or CBC, of DS captured HA-tau_151-391_ from the cell lysates (Fig. 4b–d). Almost no tau_151-391_ was captured by control brain extracts regardless of the regions (Fig. 4b, c). Thus, these results suggest higher tau seeding activity in the cerebral cortex, but no or limited tau seeding activity in the CC and CBC, of the DS brain as well as all brain regions of control cases.

### Seeding activity assessed by capture assay is correlated with tau phosphorylation

**Fig. 5:**
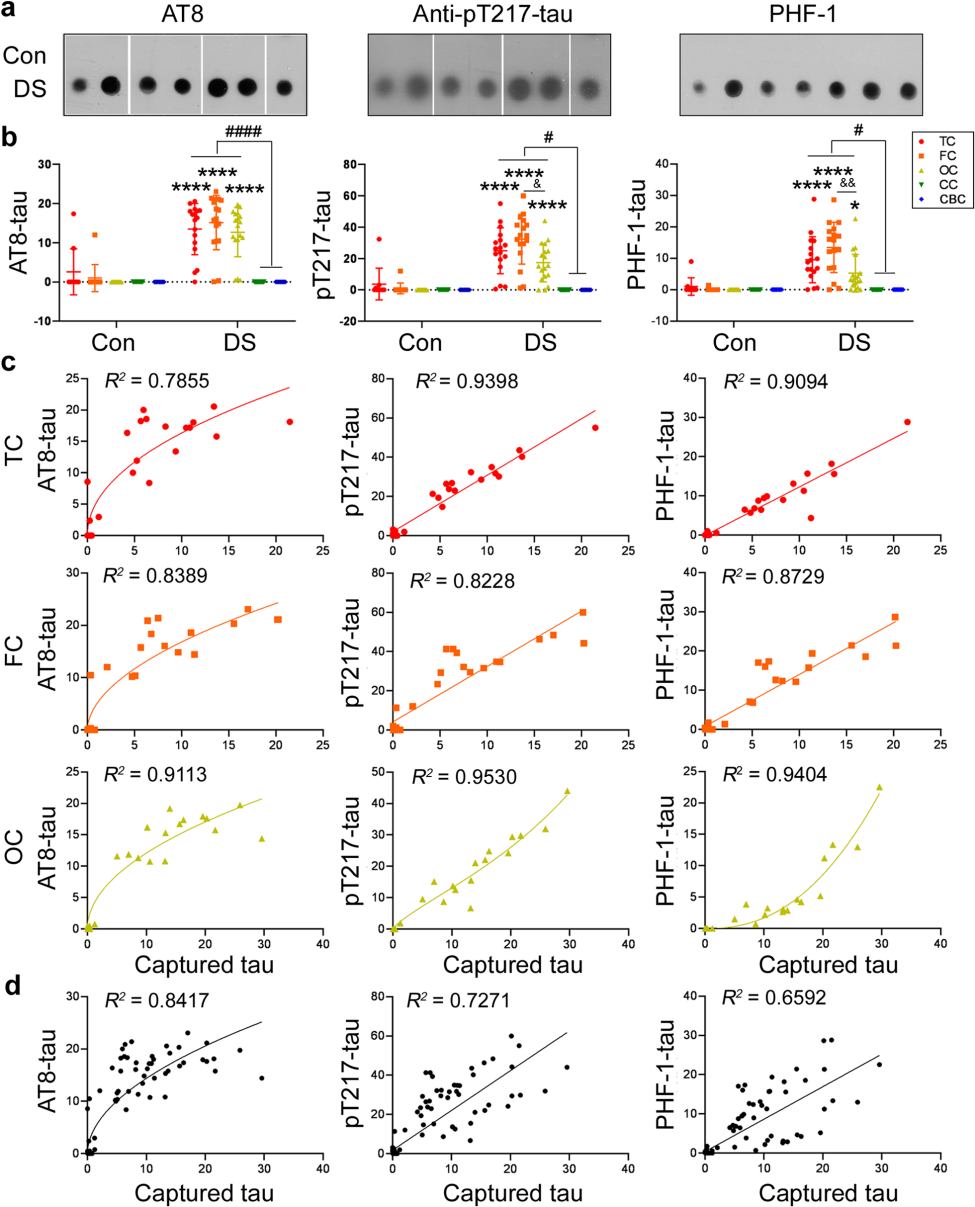
Tau seeding activity is positively correlated with the levels of hyperphosphorylated tau in DS and control brains. **a**. Representative immuno-dot blots of phosphorylated tau in the DS and control brain extracts developed with AT8 (pS202/pT205), anti-pT217-tau, and PHF-1 (pS396/404). **b**. Level of phosphorylated tau in brain extracts of temporal cortex (TC), frontal cortex (FC), occipital cortex (OC), corpus callosum (CC), and cerebellar cortex (CBC) from DS and control cases and presented as scatter plots with mean ± SD. *,^#^, or ^&^*P* < 0.05; ** or ^&&^*P* < 0.01; **** or ^####^*P* < 0.0001. **c**, **d**. Levels of phosphorylated tau at Ser202/Thr205 (AT8), Thr217, and Ser396/404 (PHF-1) in TC, FC, and OC (**c**), and all five regions (**d**) were plotted against levels of captured tau. Linear- or non-linear-regression between levels of captured tau and phosphorylated tau were performed

In DS brain, tau is hyperphosphorylated [44]. To learn the relationship between tau seeding activity and tau hyperphosphorylation, we analyzed the levels of phosphorylated tau in five regions of DS and control brains by immuno-dot blots. We found hyperphosphorylated tau at Ser202/Thr205 (AT8), Thr217, and Ser396/404 (PHF-1) in the TC, FC, and OC, but not the CC or CBC of DS brains (Fig. 5a,b). We performed linear and non-linear regression analyses between phospho-tau and tau seeding activity. We found that tau seeding activity represented by the levels of captured tau in the TC, FC, and OC of DS and control brains was positively correlated with the levels of phosphorylated tau at Ser202/Thr205, Thr217, and Ser396/404 (Fig. 5c). Furthermore, we found a positive correlation between tau seeding activity and the levels of phosphorylated tau at Ser202/Thr205, Thr217, and Ser396/404 in five brain regions of DS and control cases. Thus, tau seeding activity is positively correlated with its hyperphosphorylation.

Braak staging scores accumulation of phospho-tau based on AT8 antibody staining of specific brain regions [5]. Braak stage I was detected in the youngest, 28 years old DS subject, Stage V was detected in two 43 years old subjects whereas in all DS subjects from 47 to 74 years old the topography of neurofibrillary tangles matched stage VI. In all DS subjects topography of amyloid plaques corresponded to stage C. This pattern is similar in cohorts with prevalence of middle age and old subjects with DS [60]. To test for a relationship of Braak staging and tau seeding activity in the TC of DS subjects, we carried out a nonparametric Spearman correlational analysis and found captured tau levels by DS brain extracts were positively correlated with Braak stage (Additional file 1: Fig. S3a), while age and seeding activity did not correlate (Additional file 1: Fig. S3b). Similar levels of tau were captured by the brain extract from female and male subjects (Additional file 1: Fig. S3c), indicating gender does not significantly affect the seeding activity.

Immuno-dot blots could not display the HMW-tau species. To further confirm the relationship between the seeding activity and phosphorylation of HMW-tau, we analyzed tau phosphorylation at several phosphorylation sites in OCs with Western blots and found that tau was hyperphosphorylated and displayed SDS- and β-ME resistant HMW-tau at all detected sites, including Ser199, Thr205, Thr212, Ser214, Thr217, Thr231, Ser262, Ser396/404, and Ser422 (Additional file 1: Fig. S4a). Tau seeding activities were also positively correlated with levels of phosphorylated of tau including HMW-tau species at all detected sites (Additional file 1: Fig. S4b).

### Tau seeding activity in different regions of DS brain assessed by seeded-tau aggregation assay in cultured cells

**Fig. 6:**
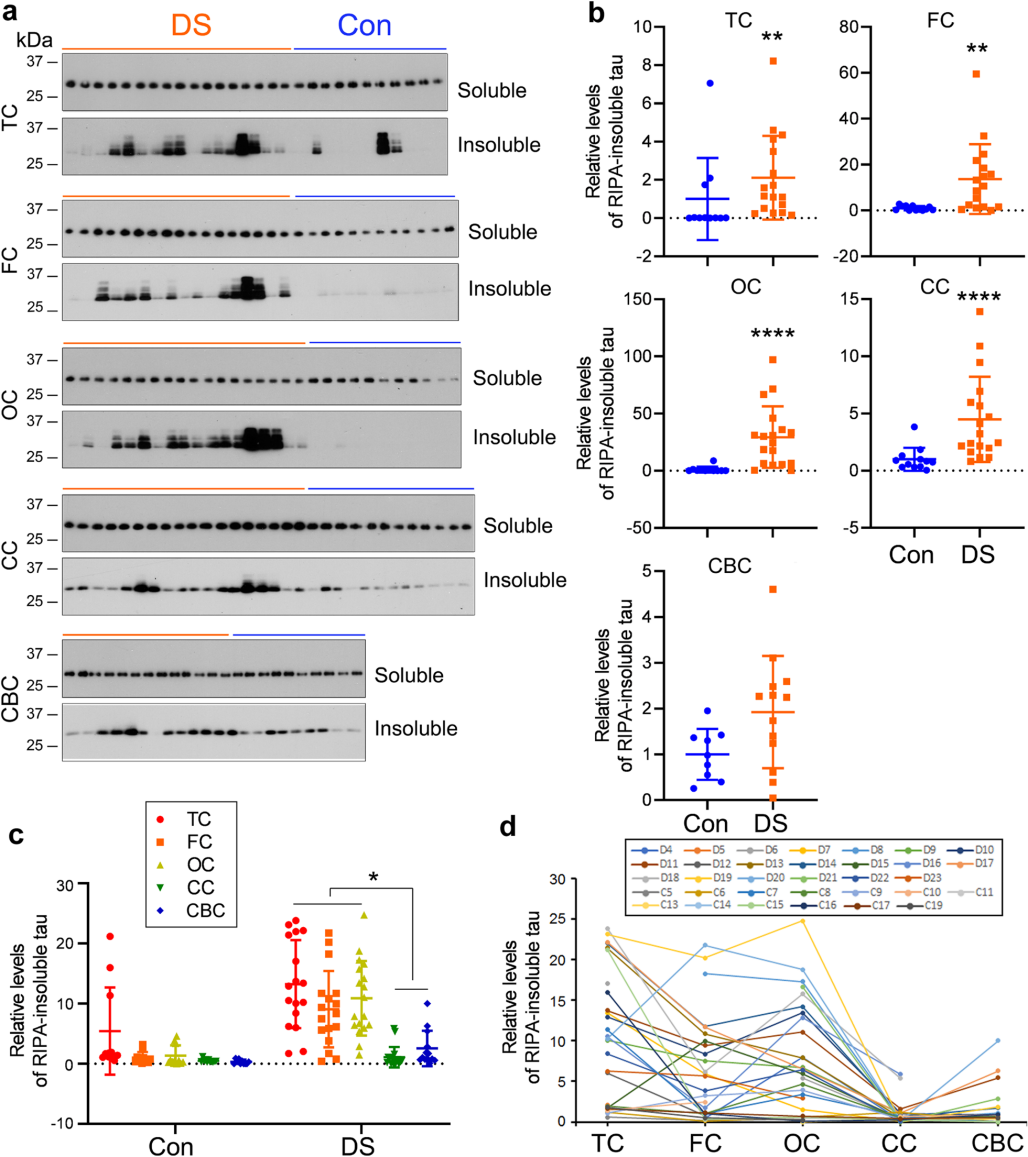
Tau seeding activity in different regions of DS brain assessed by the seeded-tau aggregation assay in cultured cells. **a**, **b** HEK-293FT cells overexpressing HA-tau_151-391_ were treated with brain extracts of various regions from DS and control cases using the same amount of tau for 42 h. RIPA-soluble and -insoluble taus were analyzed by Western blots (**a**). Levels of RIPA-insoluble tau are presented as scatter plots with mean ± SD (**b**). **c**, **d** Levels of RIPA-insoluble tau analyzed by immuno-dot blots developed with anti-HA are presented as scatter plots with mean ± SD (**c**) or as across various brain regions (**d**). *P < 0.05, **P < 0.01, ****P < 0.0001

We evaluated tau seeding activity in various DS brain regions with the seeded-tau aggregation assay in cultured cells. Because tau was less expressed in control brains and in the CC and CBC of DS, we measured tau levels in the brain extract samples with immuno-dot blots developed with a mixture of R134d and 92e as described previously [64]. HEK-293FT/HA-tau_151-391_ cells were treated for 42 h with brain extracts that contain the same levels of tau. RIPA-soluble and -insoluble taus were analyzed with Western blots. We found similar levels of RIPA-soluble tau in the cells treated with brain extracts from control and DS brains (Fig. 6a), but significantly increased RIPA-insoluble tau in the cells treated with the extracts of TC, FC, OC, and CC, but not CBC, of DS compared with corresponding regions from control cases (Fig. 6a,b), indicating tau seeding activity in DS cerebral cortices and CC but not in CBC.

To compare the seeding activity across various brain regions, we measured RIPA-insoluble tau with immuno-dot blots. We found that the levels of RIPA-insoluble tau were markedly higher in the cells treated with TC, FC, and OC than with CC and CBC extracts of DS cases (Fig. 6c), further confirming that DS cerebral cortices display higher tau seeding activity as detected above by the tau capture assay.

## Discussion

Proteopathic tau recruits monomeric tau and templates its aggregation, underlying the propagation of tau pathology in AD brain from the trans-entorhinal region to the limbic system and eventually to the primary cortical areas. Individuals with DS develop tau pathology in the 4th decade of life [62]. In the present study, we developed two assays, an in vitro tau capture assay and a seeded-tau aggregation assay in cultured cells. By using these two assays, we analyzed tau seeding activity in various regions of DS brains and found, for the first time, that the TC, FC, and CC of DS brains captured tau and seeded-tau aggregation dramatically, indicating that these three brain regions contain high tau seeding activity, whereas tau seeding activity was very limited or undetectable in the CC and CBC of DS. Tau seeding activity was positively correlated with the levels of phosphorylated tau, which displayed SDS- and β-ME resistant HMW-tau species.

Tau seeding activity in postmortem AD brain is quantitively and qualitatively correlated with disease severity and rate of progression [12]. The quantification of tau seeds in human specimens may be relevant to the clinical progression of AD and related tauopathies. In general, proteopathic seeds recruit, misfold, and template the aggregation of tau monomers, leading to their use in a wide range of seeding activity assays. So far, tau seeding activity can be quantified by in vitro seed amplification assays, such as Real-Time Quaking-Induced Conversion-based assay, RT-QuIC-based assay [12, 46], cell-based assays in cultured cells, such as FRET-biosensor assay [31], and in vivo seed amplification assays [9, 40]. Tau in AD and most other tauopathies is not mutated [22]. However, to our knowledge, almost all assays use tau with tauopathy-related mutations for seeding activity measurement. Recently, tau_151-391_ was shown to be captured and to be seeded to aggregate the most effectively by AD O-tau [26]. Here, by using HA-tau_151-391_ without tau mutation, we developed two quantitative assays, a capture assay and a seeded aggregation assay, for the detection of tau seeding activity.

The capture assay was developed on the basis of recruitment of monomeric tau by proteopathic tau seeds. Seed-competent tau was applied onto a NC membrane, and the membrane was incubated with HEK-293FT cell lysate containing HA-tau_151-391_. The recruited HA-tau_151-391_ was developed by anti-HA. Thus, the assay is low cost and practical and can be performed in a regular laboratory setting. We found that AD O-tau captured tau_151-391,_ but not TDP-43, in a dose-dependent manner. The capture ability of AD O-tau was abolished by GuHCl treatment. AD and PiD brain extracts in which tau was hyperphosphorylated and displayed SDS- and β-ME resistant HMW-tau species could capture HA-tau_151-391_ from the cell lysate, whereas control and disease brain extracts in which phosphorylated tau was undetectable could not recruit tau. Thus, capture assay is highly specific for measuring tau seeding activity. The sensitivity of capture assay was 312 ng protein of AD brain extract and ~ 1 ng tau in AD O-tau, which can be enhanced by using the enhanced ECL kit. However, this assay is much less sensitive than the RT-QulC-based assay at 2 fg tau [46], which relies on the use of heparin to promote the templating of tau substrate. Heparin induces tau aggregation effectively [20]. It was found that the structure of heparin-induced tau filaments differs from those found in AD or other tauopathies [66]. In two independent experiments, the yield of seeding activity of AD and control brain extracts was similar, indicating that the capture assay is repeatable.

The seeded-tau aggregation assay belongs to the cell-based seeding activity assay class. In this assay, HEK-293FT cells were transiently transfected to express HA-tau_151-391_ and treated with seed-competent tau by using Lipofectamine 2000 for 42 h. Ultracentrifugation of cell lysate at 10,000×*g* for 30 min yielded RIPA-insoluble tau, representing aggregated tau, and was analyzed by immunoblots. Similar to the in vitro capture assay, we found AD O-tau induced RIPA-insoluble tau aggregation dose-dependently. AD O-tau could not induce TDP-43 aggregation. AD and PiD brain extracts seeded tau_151-391_ aggregation, but not control and disease brain extracts in which phosphorylated tau was undetectable. Thus, this assay is also highly specific for measuring tau seeding activity. Two independent experiments displayed similar tau aggregation induced by the brain extracts, indicating that these assays are reliable and repeatable. The sensitivity of our seeded-tau aggregation assay was 31 ng total protein of AD brain extract, which is less sensitive than ultrasensitive FRET-biosensor assay at 153 pg to 1.2 ng of total protein from AD brain homogenates centrifugated at 21,000×*g* for 15 min [29]. Here, we used brain extracts yielded  from 10,000×*g* centrifugation for 10 min. Thus, seeded-tau aggregation assay is highly sensitive, specific, and repeatable. FRET-biosensor assay relies on the overexpression of the repeat domain (RD) of tau with the pro-aggregating P301L mutation fused to fluorescent proteins [31]. The size of the reporter fluorescent protein is two times larger than TauRD, while HA, 8 a.a. tag, is 30 times less than tau_151-391_, which is 240 a.a. We speculate that the seeded-tau aggregates may be more disease-relevant. It is known that tau aggregation relies on the microtubule binding repeats, but we do not know whether tau_151-391_ is able to fully evaluate some aggregate conformations in which N- and/or C-termini involved in. In addition, the structure of seeded tau_151-391_ aggregates remains elusive.

In addition to their specificity, sensitivity, and reproducibility, both the capture assay and the seeded-tau aggregation assay do not require special equipment and can be performed in a regular biomedical laboratory setting. The levels of captured tau were strongly and positively correlated with the levels of aggregated tau induced by the brain extracts from AD and related tauopathies. Even though these two assays are less sensitive for measuring tau seeding activity, they can be used to determine the role of post-translational modifications of tau in the captured tau and in the seeded-tau aggregates [26, 65]. Moreover, these two assays provide a platform for drug screening by targeting tau propagation.

Alternative splicing of tau exon 10 generates tau isoforms with 3R-tau and 4R-tau [8]. Its dysregulation causes several types of tauopathies. In AD, chronic traumatic encephalopathy (CTE), and several other tauopathies, all six tau isoforms are present in aggregated tau filaments. The Pick bodies of PiD are made of 3R-tau only. In PSP, CBD, argyrophilic grain disease (AGD), and several other diseases, isoforms with 4R-tau are found in the filaments [22]. By cryo-electron microscopy, the ultrastructure of tau filaments extracted from diseased brains was identified; it appears that the structures of tau filaments are distinct among diseases but identical in different individuals with AD [15], CTE [14], PiD [13], or CBD [67]. In vitro study has shown that seeds of 3R-tau and 3R/4R-tau recruit both types of isoforms, while seeds of 4R-tau recruit 4R-tau, but not 3R-tau [11]. Different RT-QuIC assays could detect specifically 3R-, 4R-, or 3R/4R-tau seeds in brain homogenates from corresponding tauopathies [46, 50]. It was found that tau aggregate propagation in cultured HEK-293T cells required isoform pairing between the infecting seeds and the recipient substrate. PiD tau aggregates seeded 3RD_VM_-YFP aggregation, whereas 4R-tau aggregates from AGD, CBD, and PSP brains induced 4RD_LM_-YFP aggregation. Tau aggregates from AD and CTE brains were unable to induce aggregation of either 3RD_VM_- or 4RD_LM_-expressing cells but were able to seed tau aggregation in HEK-293T cells expressing both 3RD_VM_-YFP and 4RD_LM_-YFP [63]. However, an in vivo study demonstrated that tau aggregates from transmission of distinct tau strains are independent of strain isoform composition, but instead intrinsic to unique pathological conformations [28]. Here, we found that AD brain extracts captured and seeded both 3R-tau_151-391_ and 4R-tau_151-391_ aggregation similarly. Unexpectedly, we also found that like AD extracts, PiD brain extract captured 3R-tau and 4R-tau and seeded their aggregation. Of note, here we used 10,000 xg extracts, but not detergent-insoluble tau, as the above cited studies used, for measuring seeding activity. The brain extract contains predominantly oligomeric tau, which is small, soluble, and freely diffusible protein assemblies that are not shaped like fibrils but are more globular [61]. It was reported that brain extracts from AD, CTE, and PiD induced 4RD_LM_-YFP aggregation in HEK-293T cells expressing high levels of 4RD_VM_-YFP [63]. Thus, we speculate that oligomeric tau has a more dynamic and loose conformation and may have less strength than filamentous tau to order isoform-matched tau aggregation. In the present study, only one of three PiD cases displayed tau hyperphosphorylation and seeding activity. Whether oligomeric tau strains have less strength in seeding strain isoform–dependent aggregation and whether these two assays can specifically detect 3R- or 4R-tau seeds remains to be investigated by using increased numbers of tauopathy cases.

Individuals with DS develop early-onset AD pathology [62]. In DS brain, tau is hyperphosphorylated at multiple sites and aggregated to form NFTs [44, 53]. The neuron-derived small extracellular vesicles from the plasma of patients with DS-AD contain phospho-tau and seed tangle-like tau pathology in mouse brain [41]. Here we analyzed regional phosphorylation of tau and found that the levels of phosphorylated tau were markedly higher in the TC, FC, and OC, but not in the CC or CBC of DS, compared with the corresponding regions of control brains. Correlating with the presence of hyperphosphorylated tau, extracts from these three cerebral cortical regions, but not from CC and CBC of DS as well as corresponding regions of control brains, captured tau, indicating DS cerebral cortex contains proteopathic tau seeds. The CC consists of axons mainly and contains less tau. However, we found increased tau aggregates seeded by CC extracts from DS than control brains, which is consistent with the findings from previous studies that AD white matter extracts seeded-tau aggregation but less potently than AD gray matter extracts did [64]. Thus, AD axons also contain proteopathic tau seeds, which may be critical for regional propagation of tau pathology. Different from the cerebral cortex, to date, no tau pathology was observed in CBC. Here, we did not find hyerphosphorylated tau or proteopathic tau seeds in DS CBC, indicating that tau pathology does not propagate in DS CBC. The postmortem interval (PMI) in the DS subjects was longer than that in AD cases used in the present study. Previously we have reported that tau is rapidly dephosphorylated in mouse brain during postmortem delay [59], but the effect of PMI on tau seeding activity remains elusive.

AD O-tau, the major proteopathic tau seeds in AD brain, is hyperphosphorylated at multiple sites [43]. Dephosphorylation passivates the seeding activity of AD O-tau in vitro [65] and in vivo [32]. Here, we found that tau seeding activity in AD and related-tauopathy brains is associated with hyperphosphorylated and HMW-tau. Although all tauopathies are characterized by tau pathology of hyperphosphorylated and seeding competent tau, we did not detect it in the tissue pieces used from several cases, suggesting that the pathology is not evenly distributed throughout the frontal cortex in tauopathies. However, we could not rule out the capability of these two assays in detecting tau seeding activity in the brain of individuals with PSP/CBD and the majority of PiD, which remains to be confirmed by recruiting more tauopathy cases to the study. In various regions of DS brain, tau seeding activity was positively correlated with the levels of hyperphosphorylated tau. However, GuHCl treatment destroyed the ability of AD O-tau to capture tau but not phosphorylation, suggesting misfolded conformation of tau is crucial for its seeding activity.

Aggregation of proteins to form amyloid relies on the β-sheet [61]. GuHCl and urea are chaotropic denaturants used in physiochemical studies of protein-folding. At high concentrations of GuHCl or urea, proteins lose their ordered structure, and they tend to become randomly coiled, i.e., they do not contain any residual structure. Here, we found 6 M GuHCl, but not 8 M urea, abolished the seeding activity of AD O-tau. GuHCl is often found to be approximately twice as efficient as urea in denaturing proteins, but this activity varies with the protein targets [24]. In addition to GuHCl and urea, heat treatment also denatures proteins. Boiling of AD O-tau does not affect phosphorylation or seeding activity of AD O-tau [42]. Here, we found that treatment with 6 N GuHCl completely inhibited the seeding activity, indicating that secondary structure, e.g., β-sheet, is essential for tau seeding activity, and the denaturing agents selectively kill tau seeding activity.

In summary, here, we report two specific, sensitive, and reproducible assays with low cost for assessing tau seeding activity, which can be used for evaluating the effect of post-translational modifications on templated tau aggregation and drug screening. By using these two assays, we found high tau seeding activity in the TC, FC, and OC; low seeding activity in the CC; and no seeding activity in the CBC of the DS brain. Tau seeding activity is highly correlated with levels of hyperphosphorylated and HMW-tau species. As in AD, propagation of tau in DS brain may underlie the development of dementia in this disease.

## Supplementary Information

**Additional file 1**. Supplementary figures.

## Data Availability

The datasets generated and/or analyzed during the present study are available from the corresponding author, Dr. Fei Liu, upon reasonable request.
