# Supplementary material for: Tau seeding activity in various regions of down syndrome brain assessed by two novel assays
Source: Acta Neuropathol Commun. 2022 Sep 5;10:132. doi: 10.1186/s40478-022-01436-2 (PMC9446852; doi:10.1186/s40478-022-01436-2)
Supplement: Supplementary file 1 — Additional file 1. Supplementary figures. [file 40478_2022_1436_MOESM1_ESM.docx]

Supplemental Figures:


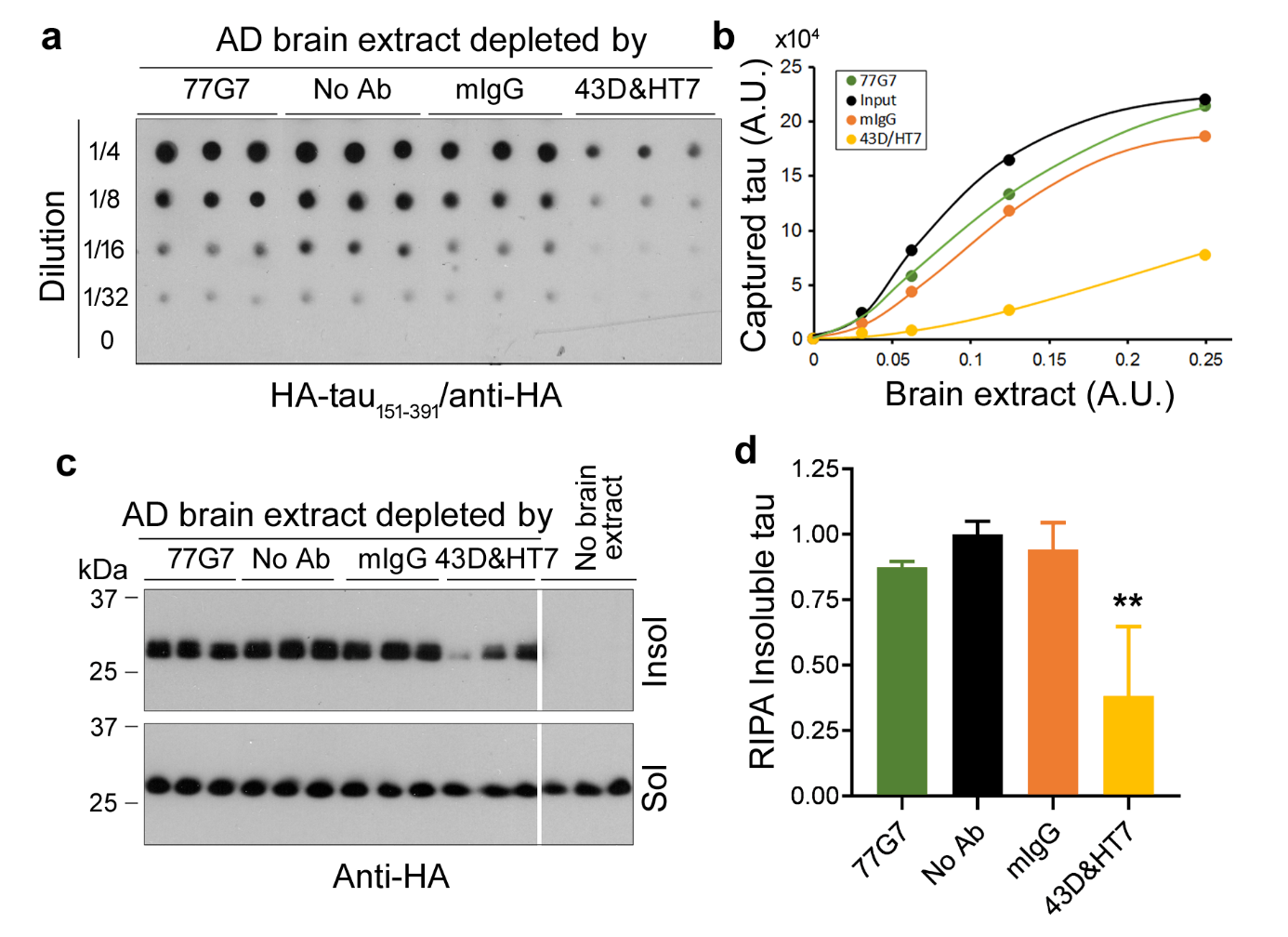


**Fig. S1** Depletion of tau reduces seeding activity of AD brain extract. **a,b** Deletion of tau with a mixture of 43D and HT7 reduced the ability of AD brain extract to capture tau. Various amounts of tau-depleted AD brain extract with various antibodies were applied onto a NC membrane. The membrane was incubated with cell extracts containing HA-3R-tau_151-391_ for capture assay (**a**). The levels of captured tau were plotted against protein amounts in the brain extracts dotted on the membrane (**b**). **c,d** Deletion of tau reduced AD brain extract to seed tau aggregation. HEK-293FT cells expressing HA-3R-tau_151-391_ were treated with same amounts of tau-depleted AD brain extract for 42 h. RIPA-insoluble and -soluble taus were analyzed by Western blots (**c**). The levels of RIPA-insoluble tau are presented as mean $\pm$ SD (**d**). **, *P* < 0.01, *vs* mIgG.


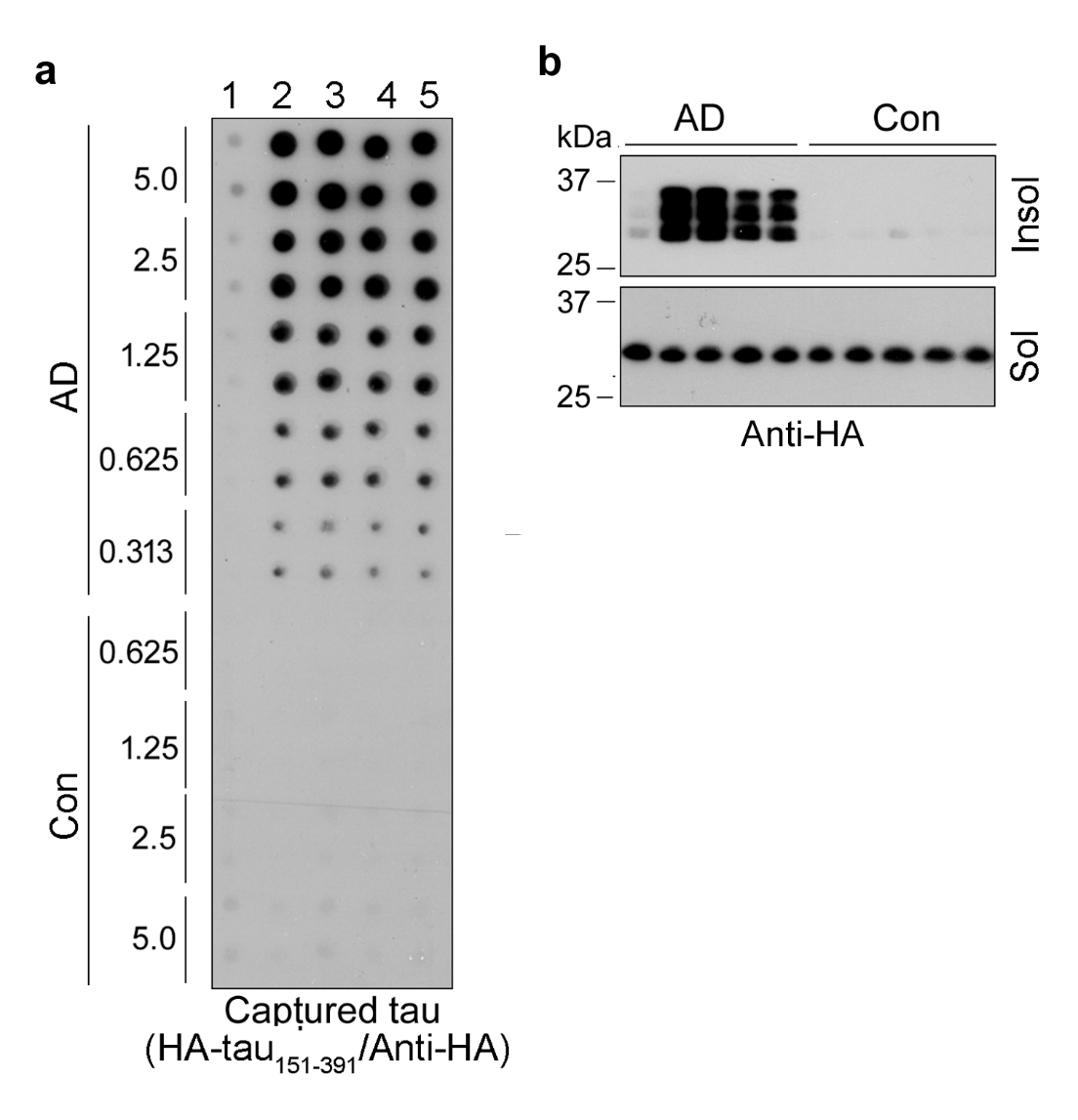


**Fig. S2** AD brain extracts capture tau_151-391_ and seed tau_151-391_ aggregation. **a** AD brain extracts captured tau_151-391_. Various amounts of brain extract from AD and control individuals were applied onto a NC membrane and incubated with HEK-293FT/HA-4R-tau_151-391_ cell extracts. Captured tau was developed with anti-HA. **b** AD brain extracts seeded tau_151-391_ aggregation. HEK-293FT cells expressing HA-4R-tau_151-391_ were treated with brain extracts from AD and control individuals for 42 h. RIPA-insoluble and -soluble taus were analyzed by Western blots developed with anti-HA.


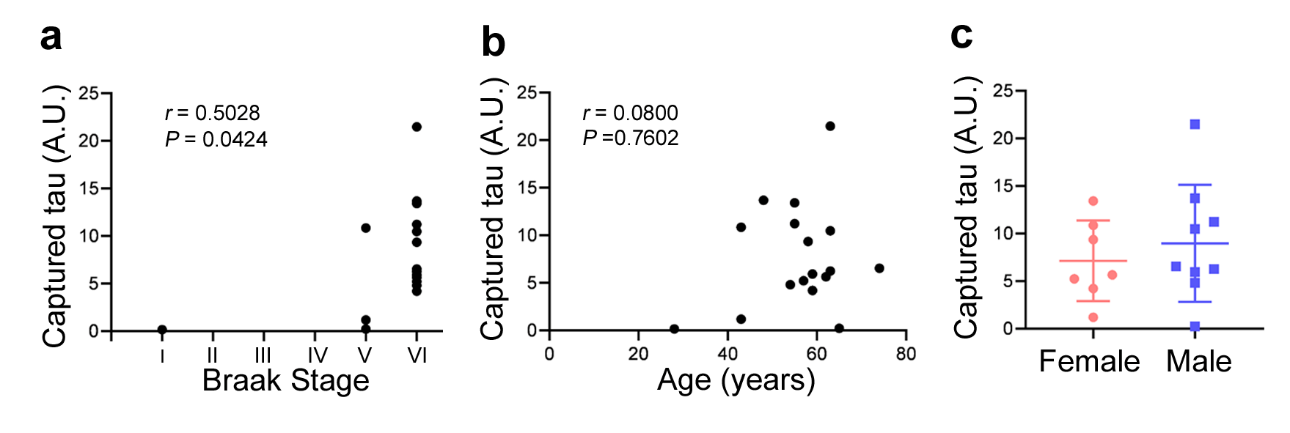


**Fig. S3** Tau seeding activity in temporal cortex of DS is positively correlated with the Braak stage. **a,b** Braak stage (a) or Age (b) was plotted against the levels of captured tau representing the seeding activity in the TC of DS. The correlation between Braak stage or age and captured tau was analyzed by using nonparametric Spearman correlational analyses. **c** Tau seeding activity represented by captured tau in the TC extract of female and male subjects of DS.


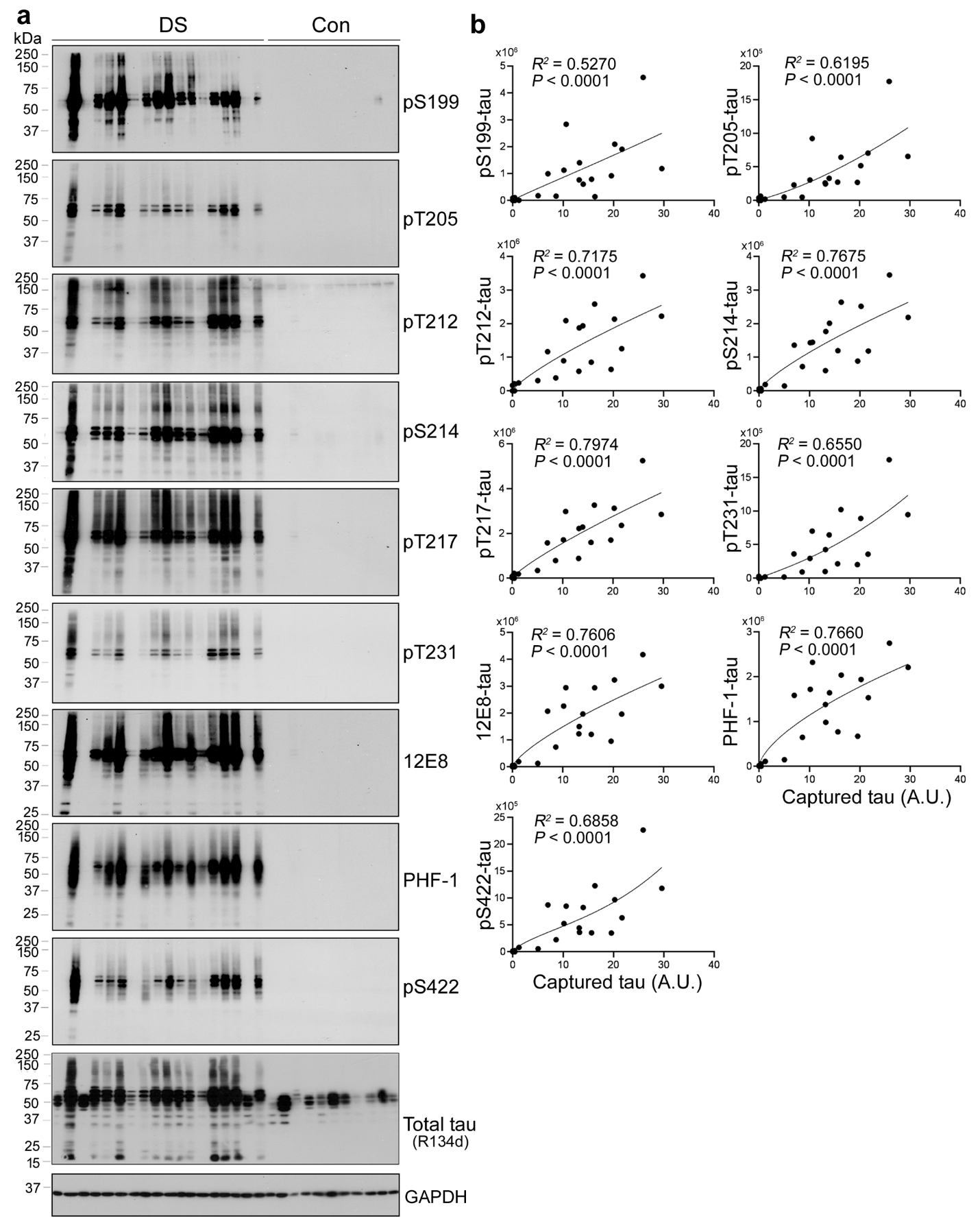


**Fig. S4** Tau seeding activity of occipital cortex is positively correlated with the level of hyperphosphorylated tau. **a** Phosphorylated tau in the OC extracts of DS and control brains were analyzed by Western blots developed with phospho-tau antibodies. **b** Levels of phosphorylated tau were plotted against the levels of captured tau representing the seeding activity. The correlation between phosphorylation and captured tau was analyzed by non-linear regression.
